# Rift Valley Fever Virus Encephalitis Is Associated with an Ineffective Systemic Immune Response and Activated T Cell Infiltration into the CNS in an Immunocompetent Mouse Model

**DOI:** 10.1371/journal.pntd.0002874

**Published:** 2014-06-12

**Authors:** Kimberly A. Dodd, Anita K. McElroy, Tara L. Jones, Sherif R. Zaki, Stuart T. Nichol, Christina F. Spiropoulou

**Affiliations:** 1 Viral Special Pathogens Branch, Division of High Consequence Pathogens and Pathology, Centers for Disease Control and Prevention, Atlanta, Georgia, United States of America; 2 University of California, Davis, School of Veterinary Medicine, Davis, California, United States of America; 3 Emory University School of Medicine, Department of Pediatrics, Atlanta, Georgia, United States of America; 4 Infectious Disease Pathology Branch, Division of High Consequence Pathogens and Pathology, Centers for Disease Control and Prevention, Atlanta, Georgia, United States of America; Aix Marseille University, Institute of Research for Development, and EHESP School of Public Health, France

## Abstract

**Background:**

Rift Valley fever virus (RVFV) causes outbreaks of severe disease in livestock and humans throughout Africa and the Arabian Peninsula. In people, RVFV generally causes a self-limiting febrile illness but in a subset of individuals, it progresses to more serious disease. One manifestation is a delayed-onset encephalitis that can be fatal or leave the afflicted with long-term neurologic sequelae. In order to design targeted interventions, the basic pathogenesis of RVFV encephalitis must be better understood.

**Methodology/Principal Findings:**

To characterize the host immune responses and viral kinetics associated with fatal and nonfatal infections, mice were infected with an attenuated RVFV lacking NSs (ΔNSs) that causes lethal disease only when administered intranasally (IN). Following IN infection, C57BL/6 mice developed severe neurologic disease and succumbed 7–9 days post-infection. In contrast, inoculation of ΔNSs virus subcutaneously in the footpad (FP) resulted in a subclinical infection characterized by a robust immune response with rapid antibody production and strong T cell responses. IN-inoculated mice had delayed antibody responses and failed to clear virus from the periphery. Severe neurological signs and obtundation characterized end stage-disease in IN-inoculated mice, and within the CNS, the development of peak virus RNA loads coincided with strong proinflammatory responses and infiltration of activated T cells. Interestingly, depletion of T cells did not significantly alter survival, suggesting that neurologic disease is not a by-product of an aberrant immune response.

**Conclusions/Significance:**

Comparison of fatal (IN-inoculated) and nonfatal (FP-inoculated) ΔNSs RVFV infections in the mouse model highlighted the role of the host immune response in controlling viral replication and therefore determining clinical outcome. There was no evidence to suggest that neurologic disease is immune-mediated in RVFV infection. These results provide important insights for the future design of vaccines and therapeutic options.

## Author Summary

Rift Valley fever virus (RVFV) is a mosquito-borne virus that causes severe disease in people and livestock throughout Africa and the Arabian Peninsula. Human disease is usually self-limiting, but a small proportion of individuals develop fatal encephalitis. The role of the host immune response in determining disease outcome is largely unknown. In order to compare the quality and character of immune responses in nonfatal and fatal cases, we used an attenuated RVFV to inoculate mice by two routes. Subcutaneous inoculation resulted in a subclinical systemic infection that was rapidly cleared due to a robust adaptive response. In contrast, intranasal inoculation stimulated weaker immune responses that failed to control virus replication and culminated in uniformly fatal encephalitis. With many encephalitic viruses, the onset of disease is mediated by changes in blood brain barrier permeability and often, subsequent injury to the CNS by an uncontrolled immune response. However, our results suggest that development of RVFV disease does not depend on either mechanism, but rather results from direct virus-mediated damage in the CNS. Future therapeutic drug design should take into account all possible routes of virus exposure as well as the role of therapies that boost the adaptive response to better combat disease.

## Introduction

Rift Valley fever virus (RVFV) is a zoonotic arbovirus that has serious public health, veterinary and economic impacts throughout Africa and the Arabian Peninsula. RVFV outbreaks are often triggered by periods of heavy rain and the subsequent emergence of RVFV-infected mosquitoes, and are characterized by the subsequent widespread abortion storms in livestock. People may become infected directly by the bite of infected mosquitoes, or by exposure to infected animal tissues. In humans, RVFV infection is generally described as a self-limiting febrile illness marked by severe arthralgia, myalgia, photophobia and headache. Although most individuals recover without long-term sequelae, some human infections progress to a serious hepatitis, hemorrhagic syndrome or delayed onset encephalitis. In these most severely afflicted patients, case fatality can be greater than 20% [1], [2], and survivors of the encephalitic syndrome frequently suffer from long-term neurological complications. Fatal RVFV encephalitic disease was first documented in the 1974–76 South African outbreak, and has since been a consistent finding in subsequent epidemics [1], [3], [4].

RVFV is a member of the family *Bunyaviridae*, genus *Phlebovirus*, and has a tripartite negative-sense RNA genome composed of a large (L), a medium (M) and a small (S) segment. The L segment encodes the RNA-dependent RNA polymerase. The structural glycoproteins Gn and Gc, as well as a nonstructural protein NSm, are encoded on the M segment. NSm is thought to function by inhibiting virus-induced apoptosis [5], [6] as well as by mediating replication in the mosquito vector [7]. The ambisense S segment encodes the nucleoprotein (N) that is required for RNA synthesis, and the nonstructural NSs protein. NSs is the major virulence factor and functions broadly by inhibiting the host immune response [8] via generalized downregulation of host transcription [9], post-transcriptional degradation of protein kinase R (PKR) [10], [11] and TFIIH p62 [12], and repression of the interferon-β (IFNβ) promoter [13].

The critical role of NSs in RVFV pathogenesis has been clearly illustrated in a number of studies, including the RVFV mouse model. Following infection with wild-type RVFV, C57BL/6J mice succumb to a fatal hepatitis within 2–3 days of infection, regardless of the dose or route of administration. In contrast, subcutaneous inoculation of a highly attenuated virus lacking NSs (ΔNSs) results in a widely disseminated infection that is cleared with no indication of clinical disease. We recently demonstrated that viral clearance and protection from clinical disease is dependent on a functional CD4^+^ T cell response. In that study, 30% of mice depleted of CD4^+^ T cells succumbed to neurologic disease following ΔNSs infection, in association with reduced antibody titers and evidence of altered CD4^+^ T cell regulatory function [14]. The results indicated that severe neurologic disease developed following a reduced systemic immune response, but also raised the question of whether neurologic disease resulted from direct viral damage to the CNS or to pathology resulting from disruption of the blood brain barrier and an unregulated immune response. To address this question, a mouse model of RVFV infection with route-dependent disease was utilized as a platform to explore the differences in the host immune response between animals that developed fatal RVFV encephalitis and those that had subclinical infections.

Here we report that intranasal (IN) infection of the highly attenuated ΔNSs virus resulted in consistently fatal neurologic disease 7–9 days after infection, in contrast to the uniformly subclinical infection that follows subcutaneous ΔNSs infection. Intranasal virus inoculation has been commonly utilized in mouse models of human viral encephalitic diseases, including Herpes simplex virus [15], influenza A virus [16], Sindbis virus [17], Venezuelan equine encephalitis virus [18]–[20], western equine encephalitis virus [21] and Hendra virus [22]. This method of inoculation can be a proxy for aerosol exposure, which has been shown to be an important route of RVFV exposure in laboratory settings [23], and is thought to play a role in the infection of individuals handling infected animal tissues. Aerosol exposure of wild-type RVFV has been shown to cause uniformly fatal disease in mice [24], [25], similar to subcutaneous infection with the same virus. Use of the ΔNSs virus model in this study permitted characterization and direct comparison of the host immune responses and viral kinetics in mice that developed fatal encephalitis (IN-inoculation) with those that had a subclinical infection (FP-inoculation). Fatal infections were associated with weak systemic immune responses and a subsequent failure to clear virus from the periphery, and onset of clinical disease correlated with high viral RNA loads and infiltration of activated T cells in the CNS. Interestingly, depletion of T cells did not improve survival, suggesting that in contrast to other encephalitic viruses [26], [27], that RVFV neurologic disease is not immune-mediated. These results provide important insights into the pathogenesis of severe RVFV infection that could inform the development of therapies targeted towards treating or preventing RVFV mediated encephalitis.

## Methods

### Ethics statement

Animal procedures in this study complied with institutional guidelines, the US Department of Agriculture Animal Welfare Act, and the National Institutes of Health Guidelines for the humane use of laboratory animals. All procedures were approved by the Centers for Disease Control and Prevention (CDC) Institutional Animal Care and Use Committee (IACUC) (Protocols 2023 and 2409).

### Mice, viruses, and biosafety

All work with infectious RVFV was completed in a biosafety level (BSL)-3E laboratory. Female 8–10-week-old C57BL/6J mice were obtained from Jackson Laboratories and were housed within BSL-3E laboratories in microisolator pans in HEPA filtration racks, following standard barrier techniques. In all animal experiments, mice were evaluated for clinical signs of disease at least once daily, and were euthanized according to a pre-determined clinical illness scoring algorithm or if found moribund.

Stocks of recombinant RVFV (strain ZH501) lacking NSs (ΔNSs) and a GFP-expressing RVFV lacking both NSs and NSm (ΔNSm/ΔNSs:GFP) were produced using reverse genetics and propagated as previously described [28], [29]. Titers of all viral stocks were determined as tissue culture infective dose 50 (TCID_50_) on VeroE6 cells and visualized by indirect fluorescent-antibody assay (IFA) using anti-RVFV hyperimmune mouse ascitic fluid (HMAF) primary antibody. Virus sequence identity was verified by full-length genome sequencing with 6-fold redundancy prior to virus use.

### Virus inoculations

For virus inoculated intranasally (IN) or subcutaneously in the left rear footpad (FP), mice were briefly anesthetized with isofluorane. For the initial LD_50_ study, mice were inoculated with 10-fold dilutions of virus, ranging from 1×10^5^ TCID_50_ to 10 TCID_50_ per nare.

In subsequent experiments, mice infected with ΔNSs IN received 1×10^4^ or 1×10^5^ TCID_50_ in 10 µL sterile Dulbecco's modified Eagle's medium (DMEM) per nare. Mice infected in the left rear FP received 2 × 10^5^ TCID_50_ in 20 µL of sterile DMEM subcutaneously. Sham-inoculated mice were given sterile DMEM. Significant differences between survival curves were analyzed using a log-rank (Mantel-Cox) test (GraphPad Prism; GraphPad Software, Inc.).

### T cell depletions

C57BL/6J mice were depleted of CD4^+^ and CD8^+^ T cells using monoclonal anti-CD4 (GK1.5) and anti-CD8 (YTS169) antibodies, or mock-depleted using an isotype control antibody (LTF2) (all obtained from Bio X Cell). Antibodies were diluted in sterile phosphate buffered saline (PBS); 300 µg of GK1.5 and 300 µg of YTS169 were administered to each mouse intraperitoneally on days -3 and -1 prior to virus infection. Depletion efficiency was determined by flow cytometry to be greater than 99%.

### Evans blue dye blood-brain barrier (BBB) permeability assay

One hour prior to euthanasia, mice were injected intraperitoneally (IP) with 800 µL of 1% Evan's blue dye. Mice were deeply anesthetized with isofluorane and perfused with 10 mL of PBS. Brains were removed and examined for evidence of dye uptake. Blue coloration of spleens, livers and kidneys were controls for effective distribution of dye.

### Serology

Serum was collected and used for RVFV anti-N IgG ELISA as described previously [30], [31]. Briefly, purified RVFV N protein or negative control LASV G1 protein was used at a concentration of 200 ng/well. Plates were blocked in blocking buffer (5% skim milk, 5% fetal bovine serum (FBS) and 0.1% Tween-20 in 1 × PBS) at 37°C for 1 h. Plates were incubated with serially diluted sera in blocking buffer for 1 h at 37°C. Plates were washed 3 times in 1 × PBS with 0.1% Tween-20 (PBST) and incubated with goat anti-mouse horseradish peroxidase (HRP 1∶10,000) (Jackson ImmunoResearch) in blocking buffer for 1 h. Plates were washed 3 times in PBST and ABTS substrate was added according to manufacturer's instructions (KPL Inc.). Reactions were stopped with the addition of 1% SDS and read at 405 nM. Absolute values obtained from negative controls were subtracted prior to analysis. Data were analyzed using 2-way ANOVA with Bonferroni post-tests (n = 3/group/time point; *p<0.05; **p<0.01; ***p<0.001) (GraphPad Prism; GraphPad Software, Inc.).

### Virus neutralization titers (VNT_100_)

Stock ΔNSm/ΔNSs:GFP virus was diluted to 100 TCID_50_ in 50 µL DMEM without FBS. Sera were heat-inactivated at 56°C for 30 min. In a 96-well plate, 1∶5, 1∶10, 1∶20, 1∶40, 1∶80, 1∶160, and 1∶320 serum dilutions were made in 50 µL DMEM. An equal volume of diluted RVFV was added to diluted sera and samples were incubated for 1 h at 37°C. A suspension of approximately 3×10^4^ VeroE6 cells was added to each well, and the plates were incubated for 72 h before visualization of GFP positive cells. VNT_100_ was defined as the highest dilution that permitted 100% neutralization of virus input. Data were analyzed using 2-way ANOVA with Bonferroni post-tests (n = 3/group/time point; *p<0.05; **p<0.01; ***p<0.001) (GraphPad Prism; GraphPad Software, Inc.).

### Preparation of splenocytes from mouse spleen

At time of euthanasia, spleens were harvested and immediately placed in sterile Roswell Park Memorial Institute (RPMI) media. Single cell suspensions were generated by passing spleens through a 70 µM mesh filter using the blunt end of a 5 cc syringe plunger. Cell suspensions were washed in RPMI and red blood cells (RBCs) were lysed using RBC Lysis Buffer according to manufacturer's instructions (Sigma-Aldrich). Cells were washed two additional times in PBS and suspended in RPMI for quantitation of total cells using a hemocytometer.

### Isolation of leukocytes from mouse brain

Whole brains were removed and immediately placed in sterile RPMI. Brains were macerated with a #10 blade and single cell suspensions were produced by passing tissue through a 70 µM filter using the blunt end of a 5 cc syringe. Cells were suspended in RPMI and layered on a 70/30 Percoll gradient in a 15 mL conical tube. Gradients were centrifuged at 800 × g for 20 minutes. The interface was taken and washed in 1x Hank's balanced salt solution (HBSS). RBCs were lysed according to manufacturer's instructions (Sigma-Aldrich). Cells were suspended in either flow buffer or RPMI for subsequent assays.

### Flow cytometry

Approximately 1×10^5^ splenocytes or brain PBMC were stained in flow buffer (PBS with 3% FBS) using 1∶200 dilutions of the following antibodies: fluorescein isothiocyanate (FITC) anti-CD4 (553651), FITC anti-CD8 (553031), phycoerythrin (PE) anti-CD3 (555275), PE anti-CD45 (553081), PE anti-CD11b (561689), allophycocyanin (APC) anti-CD69 (560689), or FITC anti-CD19 (561740), all from BD Biosciences. Staining was performed at 4°C for 20 min and then cells were washed twice in PBS before being counted. From each well, 50,000 events were collected on an Accuri Flow Cytometer (BD Biosciences). CFlow Sampler software (Accuri) was used to gate on the lymphocyte population. CD3 by CD4 or CD8 plots were then generated, a second gate was placed to delineate the CD3/4 or CD3/8 T cells, and the expression of CD69 on these cells was determined. Plot quadrants were set based upon the patterns observed in unstained cells. Specificity of staining was confirmed by the concomitant use of appropriate isotype control antibodies and fluorescence minus-one controls (all from BD Biosciences). Compensation was performed using single antibody stains. Three to five animals from each group of mice were tested independently on each day. Data were analyzed using 2-way ANOVA with Bonferroni post-tests (*p<0.05; **p<0.01; ***p<0.001) (GraphPad Prism; GraphPad Software, Inc.).

### B cell ELISPOTS

Multiscreen_HTS_ filter plates (EMD Millipore) were activated with 35% ethanol, and then washed in sterile PBS. Activated plates were coated with 1 µg/well of anti-mouse IgG or with 100 µL of inactivated RVFV virus (10^7^ TCID50/mL), and with 200 ng/well of affinity purified glutatione S-transferase (GST)-tagged RVFV N protein and allowed to bind overnight at 4°C. Plates were then washed in sterile PBS and 1×10^5^ splenocytes were placed per well with 3 fold dilutions. Cells were incubated for 24 hours at 37°C. Plates were washed with PBS and incubated with anti-mouse IgG HRP (1∶5000 in PBS) for 1 hour at room temperature and washed 5 times with PBS. Spots were developed using DAB substrate (Sigma-Aldrich) and plates were read on an ELISPOT reader using KS ELISPOT software (Zeiss). RVFV-specific antibody production is presented as relative to total antibody production. Data were analyzed using 2-way ANOVA with Bonferroni post-tests (n = 5/group/time point; *p<0.05; **p<0.01; ***p<0.001) (GraphPad Prism; GraphPad Software, Inc.).

### T cell ELISPOTS

Multiscreen_HTS_ filter plates (EMD Millipore) were activated with 35% ethanol, and then washed in sterile PBS. Activated plates were coated with anti-TNFα (BD Biosciences), anti-IL-4, anti-IL-2, or anti-IFNγ (Mabtech Inc.) capture antibodies and incubated overnight at 4°C. Plates were washed and 1×10^5^ splenocytes/well were plated in 2-fold dilutions and incubated for 24 h at 37°C. Plates were washed with PBS and incubated with biotinylated detection antibodies for 2 h at room temperature. Plates were washed and incubated with streptavidin HRP for 1 h at room temperature. After a final series of washes, spots were detected using DAB substrate (Sigma) and read on an ELISPOT reader using KS ELISPOT software (Ziess). Data were analyzed using 2-way ANOVA with Bonferroni post-tests (n = 3/group/time point; *p<0.05; **p<0.01; ***p<0.001) (GraphPad Prism; GraphPad Software, Inc.).

### Total RNA extraction

Mice were deeply anesthetized with isofluorane, terminally bled and perfused with 10 mL PBS. Specimens of olfactory bulb, cerebral cortex, cerebellum, brainstem, liver, spleen, popliteal lymph node, sciatic nerve and whole blood were collected. RNA was extracted using MagMax Total RNA Isolation kit (Ambion). With the exceptions of the sciatic nerve and popliteal lymph node, in which cases the entire structures were used, approximately 100 mg of tissue were placed directly in lysis buffer and homogenized using a high-throughput tissue grinder (GenoGrinder2000). Approximately 50 µL of whole blood was added directly to lysis buffer with isopropanol. Homogenates were extracted using the MagMax Express-96 Magnetic Particle Processor (Ambion) according to manufacturer's directions including a DNase treatment step.

### RVFV qRT-PCR

Liver, spleen, sciatic nerve, popliteal lymph node, olfactory bulb, cerebrum, cerebellum, brainstem and whole blood RNA were tested by RVFV qRT-PCR as described previously, using 1 µL of total RNA [29], [32], [33]. To normalize between samples, 1 µL of total RNA was used for a ribosomal RNA qRT-PCR assay (ABI Biosciences). Reactions were run on an ABI 7500 quantitative PCR machine (ABI Biosciences). Data were analyzed using 2-way ANOVA with Bonferroni post-tests (n = 5/group/time point; *p<0.05; **p<0.01; ***p<0.001) (GraphPad Prism; GraphPad Software, Inc.).

### Inflammatory cytokine gene expression qRT-PCR

RNA extracted from the cerebrum of IN or FP-inoculated mice was used to determine inflammatory gene expression. Primer probe sets for IL-6, TNFα, CCL2 and CCL5 (Applied Biosciences) were used with Superscript III Platinum qRT-PCR reagents (Invitrogen). Samples were run in 50 µL reactions: 1.5 µL of primer probe mix was combined with 25 µL Master Mix, 1 µL Taq enzyme mix, 21.5 µL sterile water and 1 µl of total RNA. To normalize between samples, 1 µL of total RNA was used for a GAPDH qRT-PCR assay (ABI Biosciences). Reactions were run on an ABI 7500 quantitative PCR machine (ABI Biosciences). Relative fold changes between mock-infected and IN- or FP-infected animals were determined using the comparative CT method as described by Schmittgen and Livak [34]. Data were analyzed using 2-way ANOVA with Bonferroni post-tests (n = 5/group/time point; *p<0.05; **p<0.01; ***p<0.001) (GraphPad Prism; GraphPad Software, Inc.).

### Histopathology and immunohistochemistry

Whole brains from mice inoculated IN with ΔNSs virus were euthanized after development of severe neurologic signs 7 and 8 dpi. Brains were fixed by immersion in 10% neutral buffered formalin for 7 days. Tissues were processed, paraffin-embedded, and sectioned following routine methods, and stained with hematoxylin and eosin (H&E) for histological examination. Immunohistochemistry (IHC) assays were performed using a polymer-based indirect immunoalkaline phosphatase detection system with colorimetric detection of antibody/polymer complex with Fast Red Chromogen (Thermo Fisher Scientific) as previously described [35]. Pre-treatment of tissue sections was applied as needed using Proteinase K (PK) (Roche, Mannheim, Germany) or Antigen Retrieval (AR) (Biocare Medical) prior to IHC staining. Tissues were evaluated using a polyclonal (PK, 1∶1000) and a monoclonal (PK, 1∶500) Rift Valley Fever virus antibody provided by the CDC (SPB). In addition, tissues were evaluated using CD68 (PK, 1∶100, DAKO Cytomation), CD3 (AR, 1∶100, Dako Cytomation), CD8 (AR, Ready-to-Use, Leica Microsystems, UK) and CD20 (PK, 1∶200, Dako Cytomation) cellular markers. Appropriate positive and negative control serum and tissues were tested in parallel for each assay.

## Results

### Intranasal inoculation with ΔNSs virus resulted in neurologic disease and mortality 7–9 dpi

**Figure 1:**
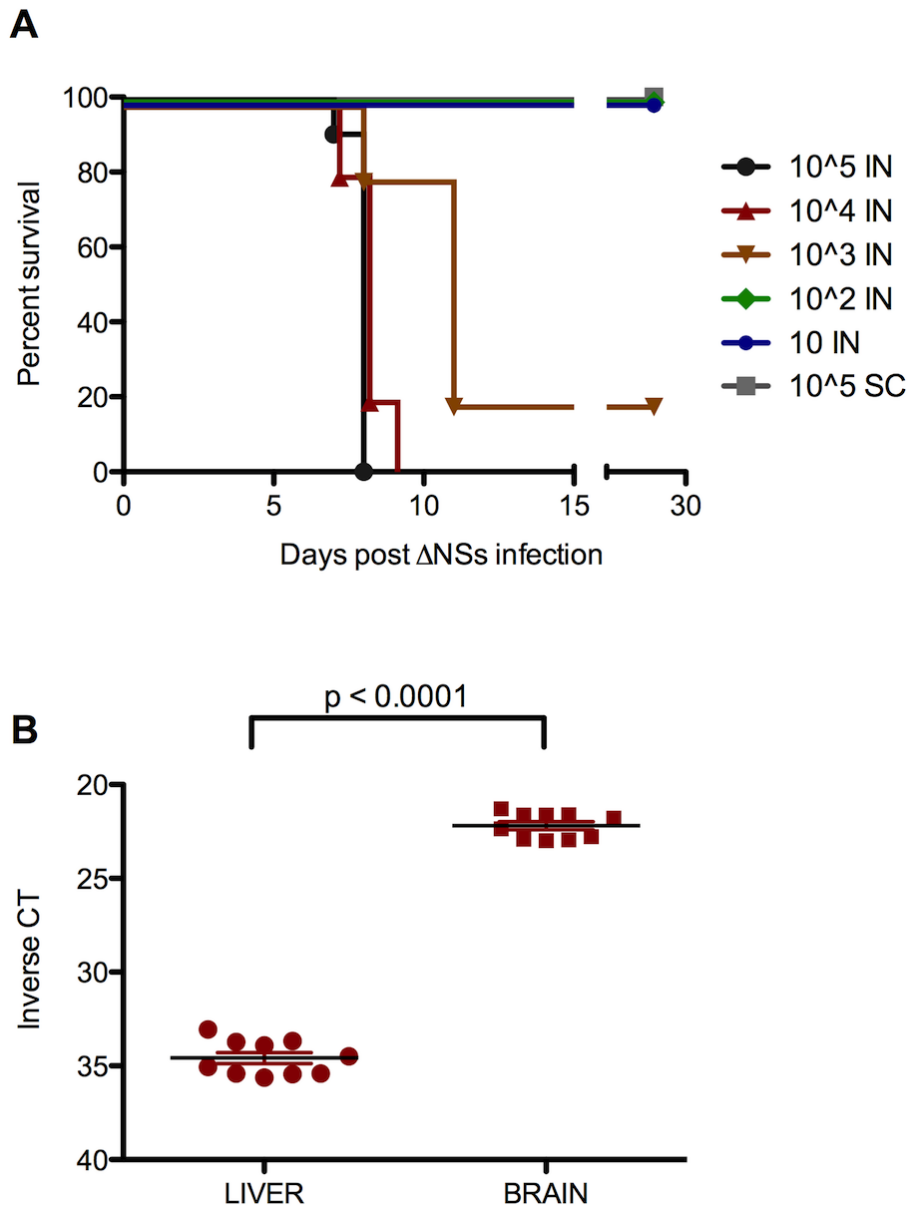
Intranasal inoculation results in fatal encephalitic disease. C57BL/6 mice were inoculated with various doses of Rift Valley fever virus (RVFV) lacking the NSs gene (ΔNSs) intranasally (IN) or subcutaneously in the footpad (FP). (A) FP-inoculated mice survived with no indication of clinical signs. IN-inoculated mice developed dose-dependent disease. Survival curves were significantly different (p<0.0001). (B) All mice that succumbed to ΔNSs virus IN infection at high doses (1×10^4^ or 1×10^5^ TCID_50_/per nare) had significantly higher viral RNA loads in the brain than in the liver, as measured by RVFV-specific qRT-PCR (p<0.0001).

As described previously [14], subcutaneous FP inoculation of 2.0×10^5^ TCID_50_ ΔNSs virus resulted in uniform survival with no indication of clinical signs, whereas mice inoculated with ΔNSs virus IN developed dose-dependent clinical disease (p<0.0001; Figure 1A). Mice infected with the highest virus doses, 2.0×10^5^ and 2.0×10^4^ TCID_50_, displayed signs of severe neurologic disease and consistently succumbed to infection 7–9 dpi (Figure 1A). At the time of death, IN-inoculated mice had significantly higher virus RNA loads in the brain relative to the liver (p<0.0001; Figure 1B). Of the mice given 2.0×10^3^ TCID_50_, 80% succumbed to infection with a prolonged time to death. Lower doses did not result in clinical disease. The striking differences between intranasal and subcutaneous inoculation provided a unique platform to characterize and compare the immune responses elicited during fatal and nonfatal infections, respectively.

### IN-infected mice rapidly developed high viral RNA loads in the brain

**Figure 2:**
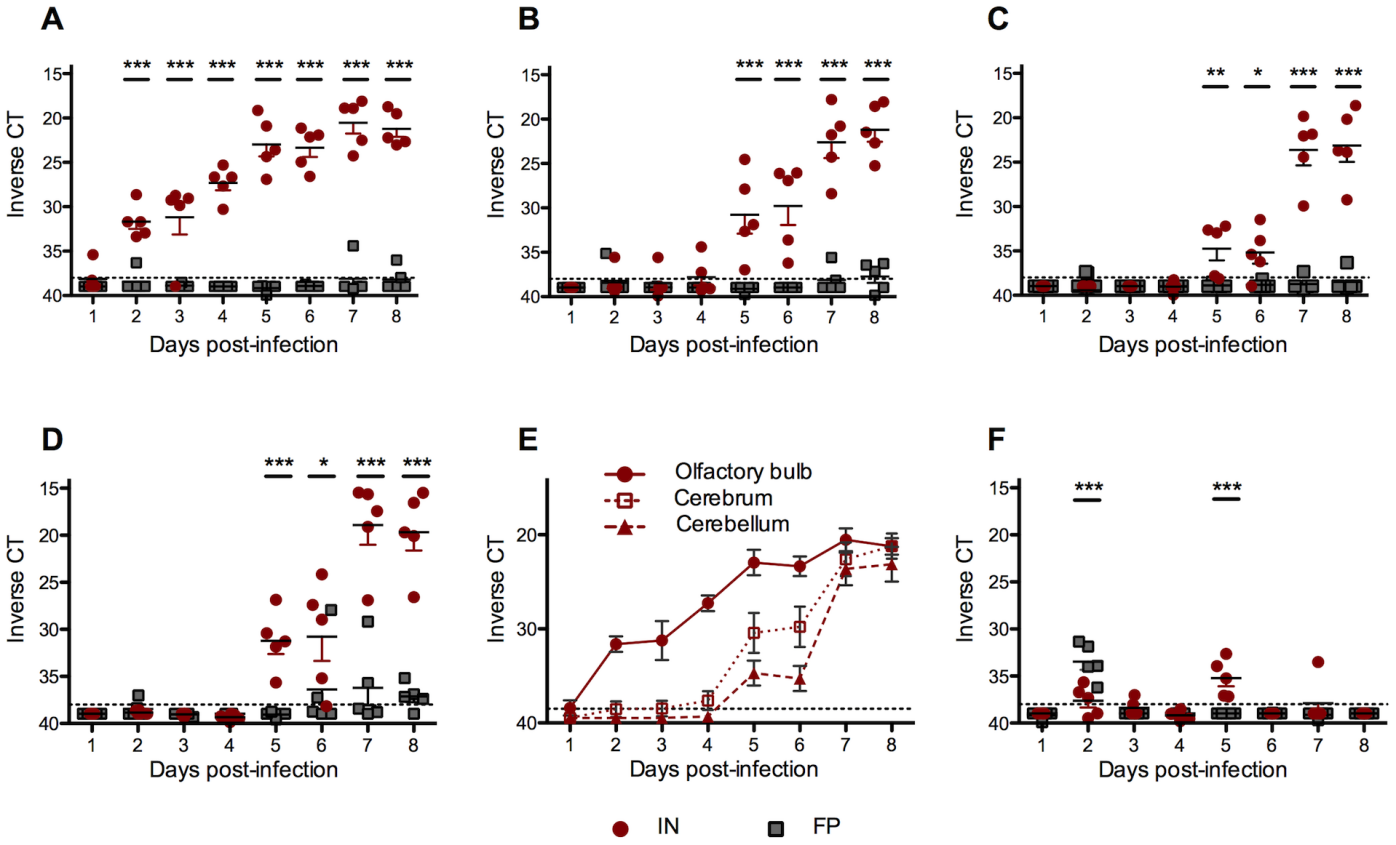
In IN-inoculated mice, virus replication was first apparent in the olfactory bulb 2 dpi and spread caudally, whereas FP-inoculated mice had low levels of virus RNA present in the brainstem at late time points. Viral RNA loads from 100(A) olfactory bulb, (B) cerebrum, (C) cerebellum, and (D) brainstem were quantified by RVFV qRT-PCR and the CT was displayed on an inverted scale. CT cut-off of 40 noted by gray dashed line. (E) The temporal pattern of virus spread in the CNS of IN-inoculated mice. (F) Viral RNA loads from sciatic nerve. Data represents vRNA loads of 5 mice per group each day.

To determine the kinetics of virus spread within the nervous system, 5 FP- and 5 IN-inoculated mice were euthanized, perfused with PBS and samples of olfactory bulb, cortex, cerebellum, brainstem and sciatic nerves were taken for RVFV-specific qRT-PCR on 1, 2, 3, 4, 5, 6, 7 and 8 dpi. The olfactory bulbs of all IN-inoculated mice tested positive for virus RNA 2 dpi and viral RNA loads steadily increased over time (Figure 2A). Subsequently, virus RNA was found in the cerebrum (Figure 2B), cerebellum (Figure 2C) and brainstem (Figure 2D). In contrast, small numbers of FP-inoculated mice had low levels of virus RNA present throughout the brain at late points in infection but these virus RNA loads were significantly lower than IN-inoculated mice. To better illustrate the temporal pattern of virus spread in the CNS of IN-inoculated mice, the virus RNA data from the olfactory bulb, cerebrum and cerebellum are presented together (Figure 2E).

All FP-inoculated mice had significant virus RNA levels present in the sciatic nerve 2 dpi, whereas all IN-inoculated mice had RVFV-positive sciatic nerves 5 dpi (Figure 2F). These data are consistent with both the route of infection (FP-inoculated mice) and with the dissemination of virus throughout the nervous system (IN-inoculated mice).

### RVF virus RNA loads were significantly higher in peripheral tissues of FP-versus IN-inoculated mice early in infection, but levels were indistinguishable after 3 dpi

**Figure 3:**
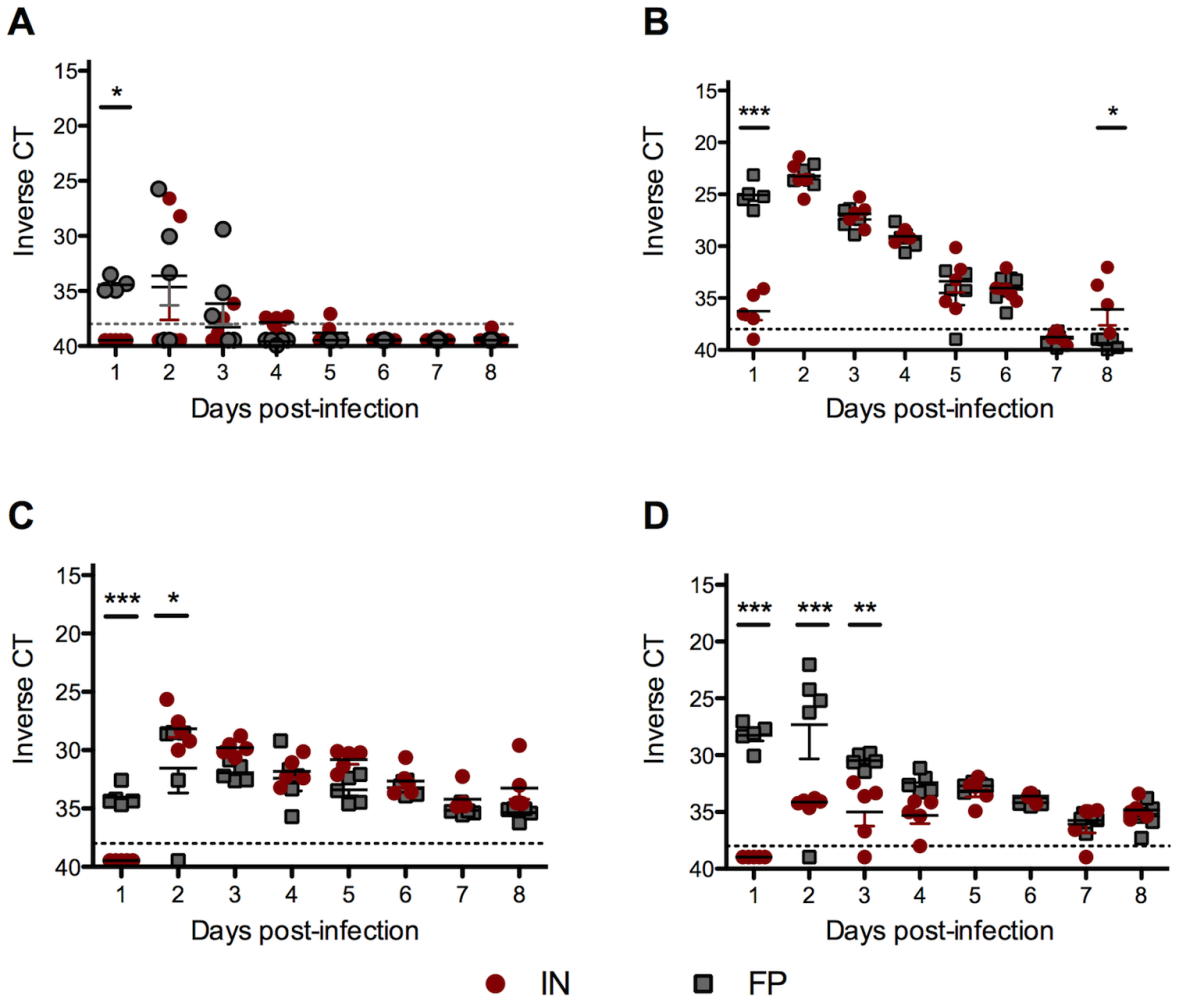
FP-inoculated mice successfully cleared ΔNSs virus despite high virus RNA levels at early timepoints. Viral RNA loads in (A) blood, (B) liver, (C) spleen and (D) draining popliteal lymph node. Averages based on 5 mice per group per time point.

In order to determine the effect of the route of infection on peripheral virus kinetics *in vivo*, we measured viral RNA loads by qRT-PCR in the blood, liver, spleen and popliteal lymph nodes. Viral RNA levels were significantly higher in the blood, liver and spleen of FP- versus IN-inoculated mice 1 dpi (Figures 3A, 3B and 3C, respectively). However, by 3 dpi, blood, liver and spleen viral RNA loads were indistinguishable between the FP- and IN-groups. FP-inoculated mice cleared virus from the liver by 7 dpi but IN-inoculated mice were unable to control virus replication in the liver. FP-inoculated mice had significantly higher virus RNA loads in the (draining) popliteal lymph node than IN-inoculated mice 1–3 dpi (Figure 3D), consistent with the route of infection.

### IN-inoculated mice produced lower levels of anti-N IgG and neutralizing antibodies than FP-inoculated mice

**Figure 4:**
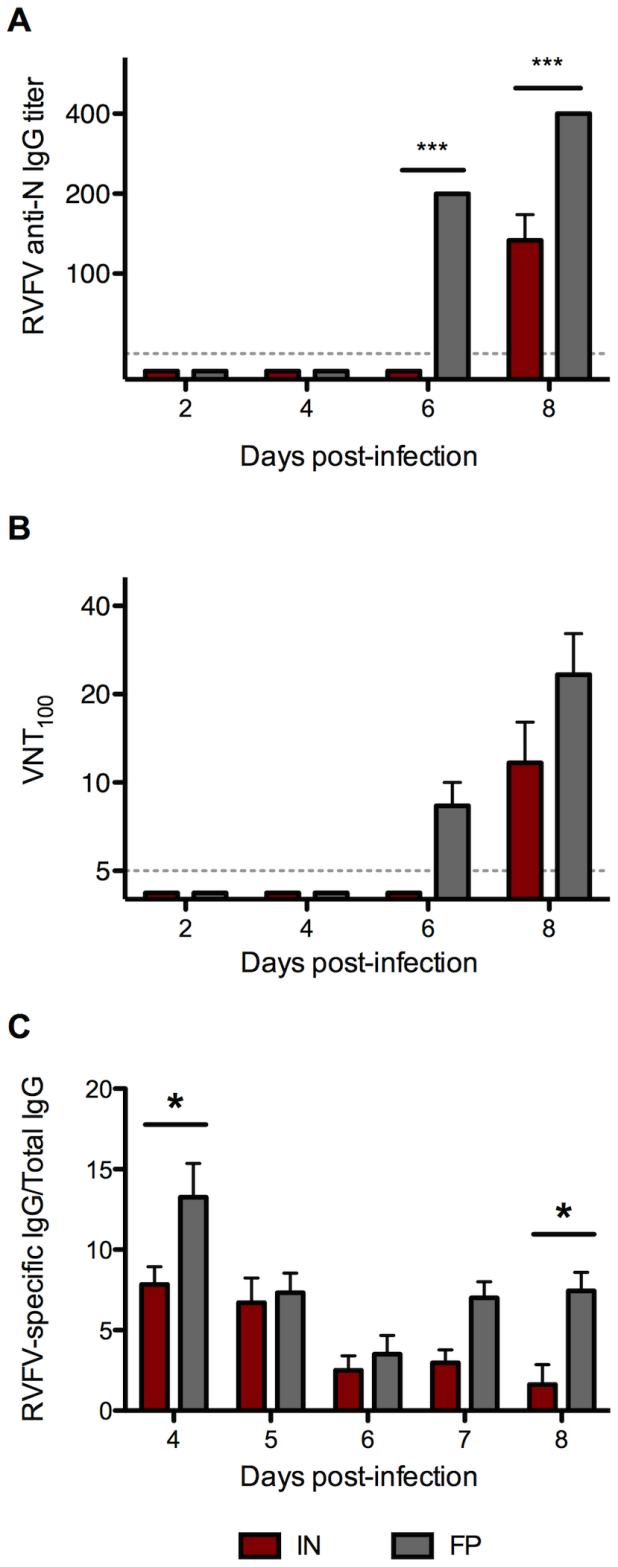
FP-inoculated mice had a significantly stronger humoral response following ΔNSs virus infection than IN-inoculated mice. Serum samples were assayed for (A) RVFV anti-N IgG antibody titers and (B) RVFV neutralizing antibody titers (averages based on 3 mice per group). Splenocytes were analyzed for (C) RVFV-specific B cells as determined by ELISPOT (averages based on 5 mice per group).

To explore differences in humoral immunity following IN or FP inoculation, we compared RVFV anti-N IgG and neutralizing antibody levels and the number of RVFV-specific B cells between groups on 2, 4, 6 and 8 dpi. Production of IgG and neutralizing antibodies were delayed by 48 hours in the IN-inoculated mice relative to the FP-inoculated mice. FP-inoculated mice had significantly higher IgG titers 8 dpi, the time at which antibody was first apparent in the IN group (Figure 4A). Only FP-inoculated mice had a detectable neutralizing antibody response 6 dpi, and higher levels 8 dpi than IN-inoculated mice (Figure 4B). FP-inoculated mice also had significantly higher numbers of RVFV-specific IgG-secreting B cells than IN-inoculated mice 4 and 8 dpi (Figure 4C).

### IN-inoculated mice had significantly more activated CD4^+^ and CD8^+^ T cells in the spleen than FP-inoculated mice

**Figure 5:**
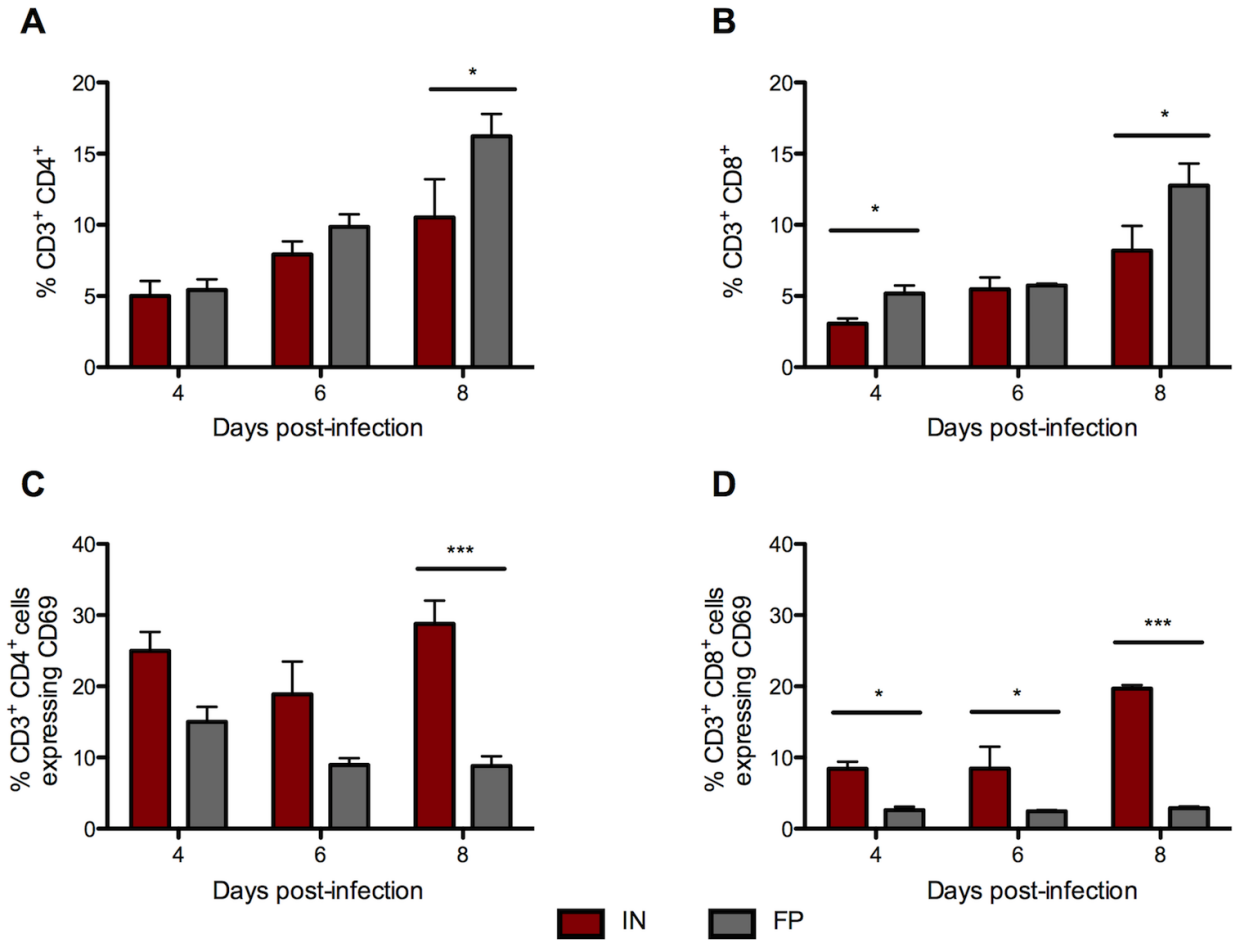
IN-inoculated mice have a significantly higher percentage of activated T cells in the spleen than FP-inoculated mice. Flow cytometry was used to determine the proportion of (A) CD3^+^/CD4^+^ and (B) CD3^+^/CD8^+^ T cells in the spleens of IN- and FP-inoculated mice. The proportion of T cells that stained positive for activation marker CD69 are shown in (C) CD3^+^/CD4^+^/CD69^+^ and (D) CD3^+^/CD8^+^/CD69^+^.Averages based on groups of 3 mice per time point.

As a broad measure of T cell activation, we next evaluated the relative proportion of CD69^+^ T cells in the spleen following IN or FP inoculation. The spleens of FP-inoculated mice had a significantly higher percentage of CD3^+^/CD4^+^ T cells 8 dpi than IN-inoculated mice (Figure 5A). The percentage of CD3^+^/CD8^+^ T cells was also significantly higher in FP-inoculated mice 4 and 8 dpi (Figure 5B). However, CD4^+^ T cell activation, as measured by expression of CD69, was higher in IN-inoculated mice than FP-inoculated mice throughout the course of infection and significantly greater 8 dpi (Figure 5C). A similar trend was apparent in CD69-positive CD8+ T cells; a higher percentage of CD8+ T cells of IN-inoculated mice expressed CD69 on all 3 days examined (Figure 5D).

### Splenocytes of FP-inoculated mice produced significantly higher levels of inflammatory cytokines

**Figure 6:**
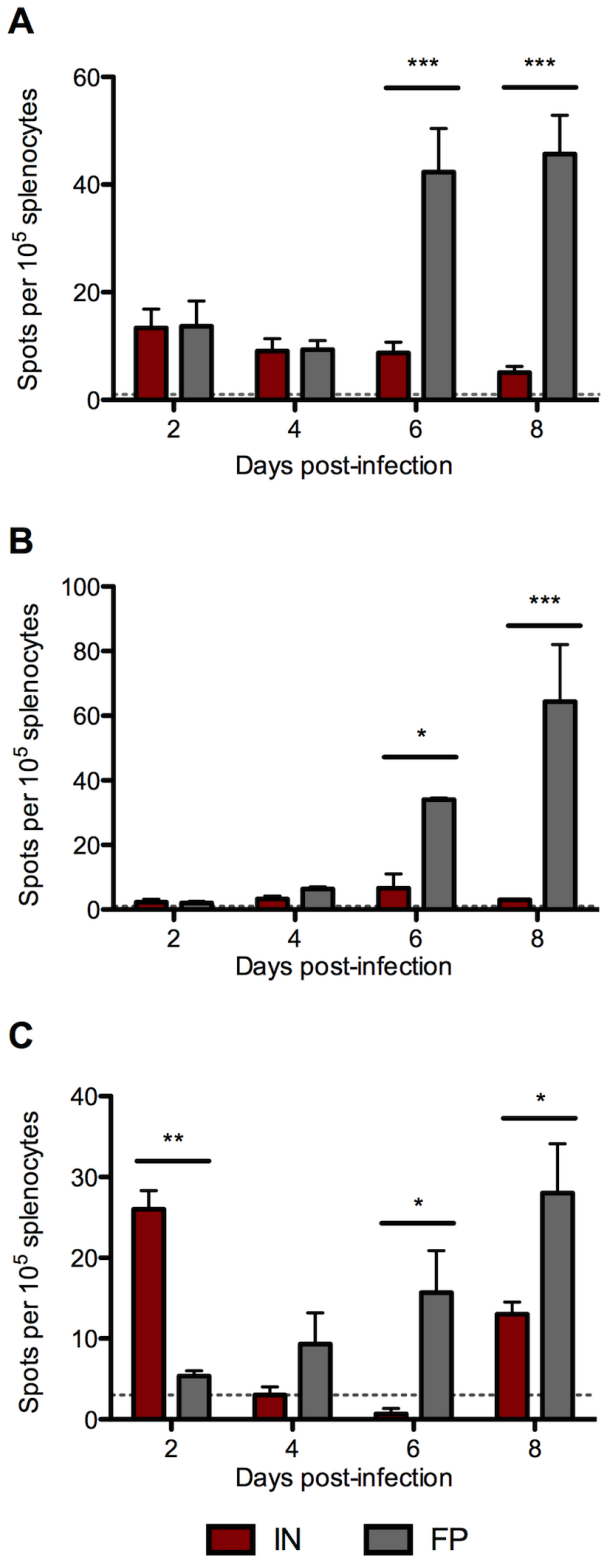
T cells from FP-inoculated mice mount a robust proinflammatory response in the spleen. Splenocytes were prepared from IN- and FP-inoculated mice and used in ELISPOT assays for proinflammatory cytokines including (A) IFNγ, (B) TNFα, and (C) IL-2. Means based on groups of 3 mice per time point.

To evaluate the quality of the cellular immune response, we measured IFNγ, TNFα and IL-2 expression in splenocytes using ELISPOT assays. Significantly more T cells from FP-inoculated mice expressed IFNγ and TNFα 6 and 8 dpi (Figures 6A and 6B, respectively). FP-inoculated mice also had more T cells producing IL-2 6 and 8 dpi, but IN-inoculated mice had higher numbers of IL-2 expressing T cells 2 dpi (Figure 6C).

### IN-inoculated mice have significantly more leukocytes, and specifically activated T cells, infiltrating the CNS following ΔNSs virus infection

**Figure 7:**
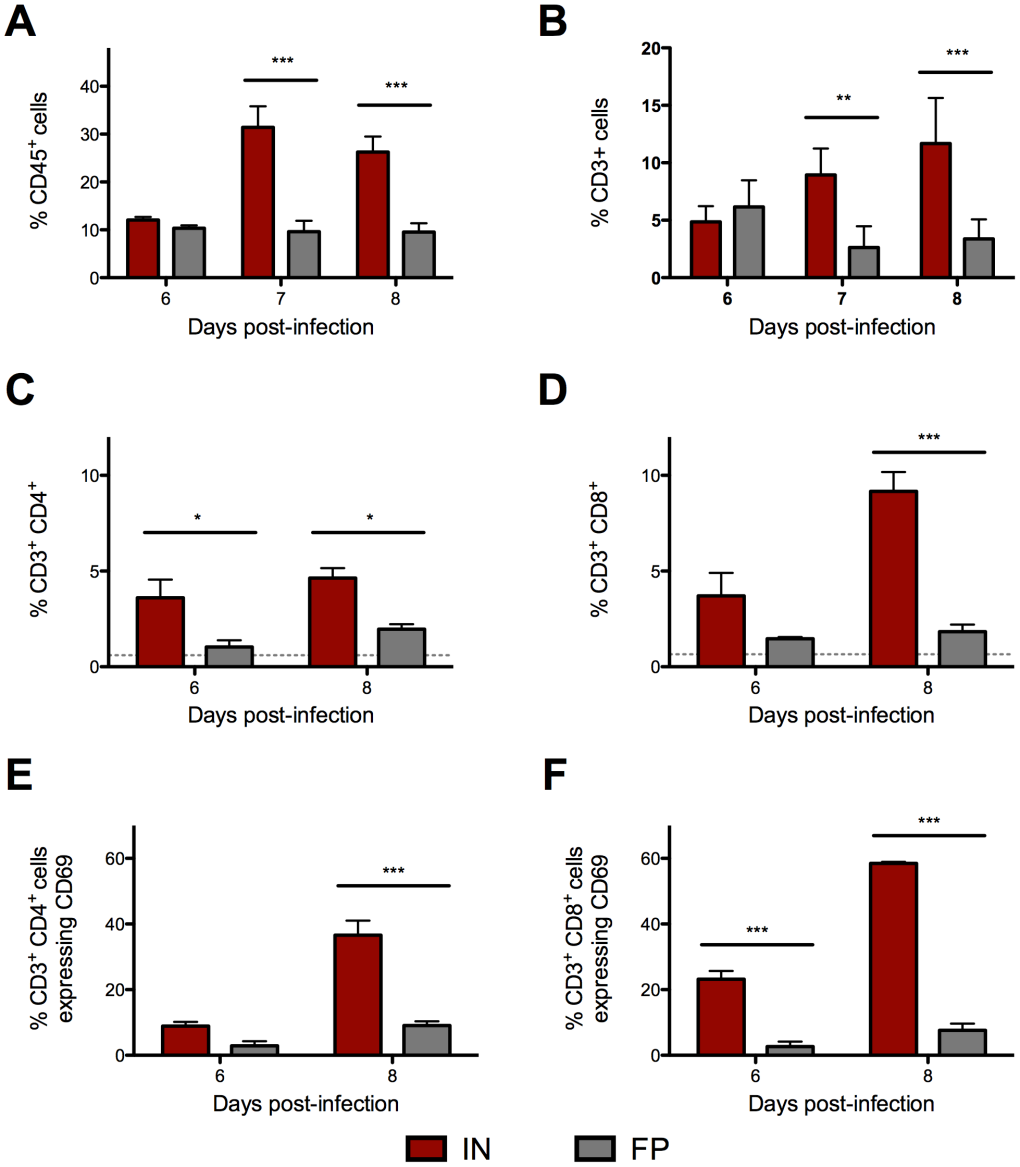
More activated CD69+ T cells infiltrated the brains of IN- than FP-inoculated mice. Leukocytes were isolated from the brains of IN- and FP-inoculated mice. In an initial experiment, of the proportion of (A) CD45^+^ cells that were (B) CD3^+^ T cells were determined. In a follow-up experiment, the proportion of (C) CD4^+^ and (D) CD8^+^ T cells in the brains of IN-inoculated mice and FP-inoculated mice were determined. The proportion of T cells that stained positive for activation marker CD69 are shown in (E) CD3^+^/CD4^+^/CD69^+^ and (F) CD3^+^/CD8^+^/CD69^+^.Means based on groups of 3 mice per time point.

Leukocyte migration into the CNS was compared between groups using flow cytometry. In an initial experiment, IN-inoculated mice had a significantly higher proportion of CD45^+^ cells than FP-inoculated mice 7 and 8 dpi (Figure 7A), and the majority of these appeared to be T cells given positive staining for CD3 (Figure 7B). In a subsequent experiment, leukocytes were harvested from brains of IN- and FP-inoculated mice late in infection, 6 and 8 dpi. In IN-inoculated mice, there were significantly more CD4^+^ (Figure 7C) and CD8^+^ (Figure 7D) T cells, than in FP-inoculated mice. In brains from the IN group, a significantly higher percentage of CD4^+^ T cells were expressing CD69 (Figure 7E). Similarly, on 6 and 8 dpi, a striking number of CD8^+^ T cells were also CD69^+^ in the brains of IN-inoculated mice (Figure 7F).

### RVFV infection did not alter BBB permeability

Given the early neuroinvasion and increased numbers of leukocytes seen in the brains of IN-inoculated mice, we sought to determine if RVFV infection resulted in an increase in BBB permeability. 5 FP- and 5 IN-inoculated mice were injected with Evans blue dye one hour prior to euthanasia on 1, 2, 3, 4, 5, 6, 7 and 8 dpi. Mice were then anesthetized and completely perfused with PBS, and whole brains were removed. No blue dye coloration was seen in the brains from FP- or IN-inoculated mice at any time point (data not shown) indicating that there was no breakdown of the blood brain barrier during infection. Blue coloration of liver, spleen and kidneys was apparent in all mice, indicating adequate dye perfusion.

### IN-infected mice have significantly higher proinflammatory cytokine expression in the brain than FP-inoculated mice

**Figure 8:**
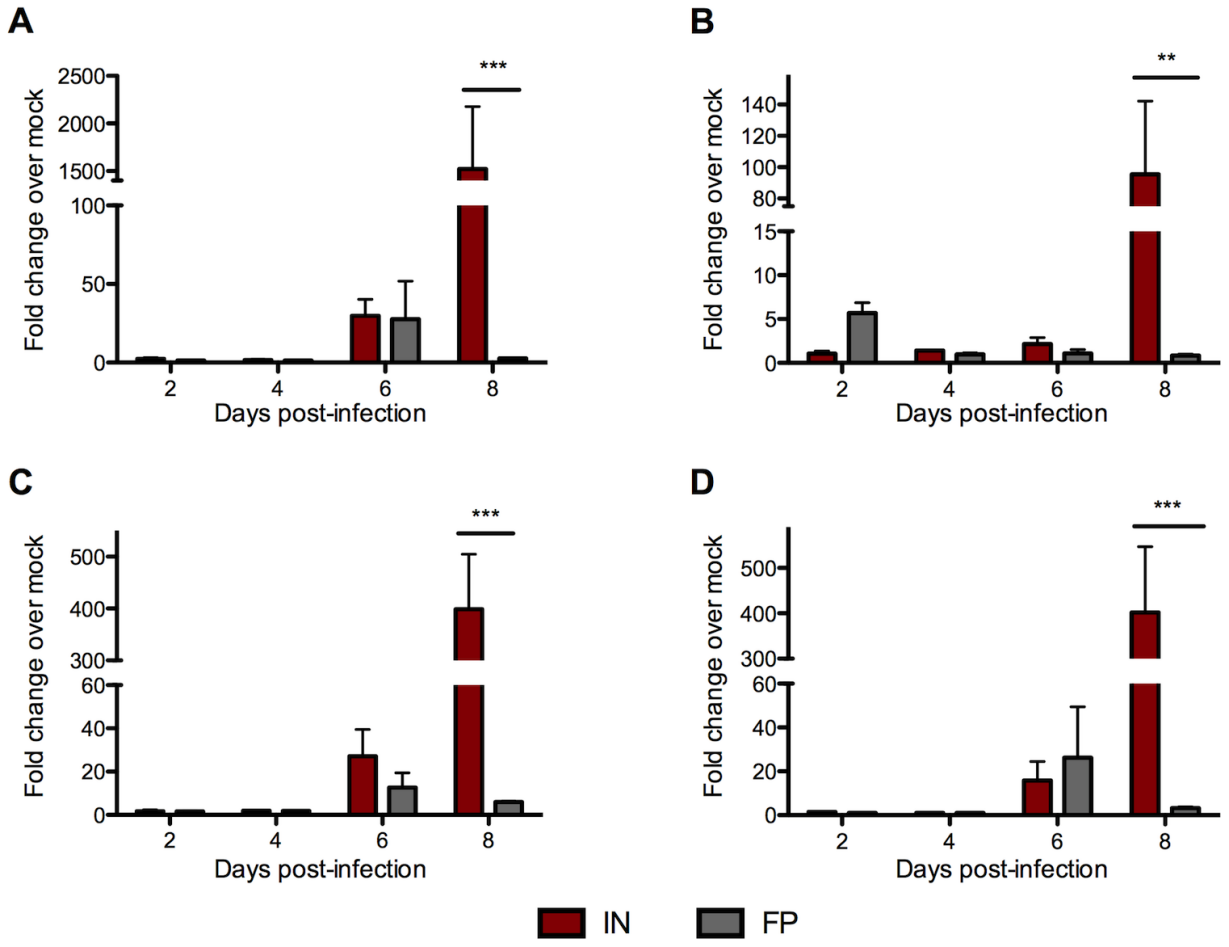
IN-inoculated mice displayed a strong proinflammatory cytokine response in the brain 8 dpi. Gene expression of several proinflammatory cytokines in the cerebrum of IN- and FP-inoculated mice was quantified by qRT-PCR, including (A) CCL2, (B) CCL5, (C) IL-6, and (D) TNFα. A housekeeping gene (GAPDH) was used to normalize gene expression data between all samples. Relative fold change was calculated by comparing expression in infected (IN or FP) tissues with mock-infected samples. Means based on groups of 5 mice per time point.

To gain a more detailed understanding of the inflammatory response in the CNS, we evaluated inflammatory gene expression in the brains of IN- and FP-inoculated mice. We found that both groups demonstrated a mild increase in expression of CCL2, IL-6 and TNFα 6 dpi. However mice inoculated IN, but not FP, displayed significant upregulation of several key inflammatory cytokines 8 dpi, including CCL2, CCL5, IL-6 and TNFα, (Figure 8 A-D) that coincided with the development of severe clinical disease.

### IN-infected mice had evidence of widespread viral infection throughout the CNS at late time points

**Figure 9:**
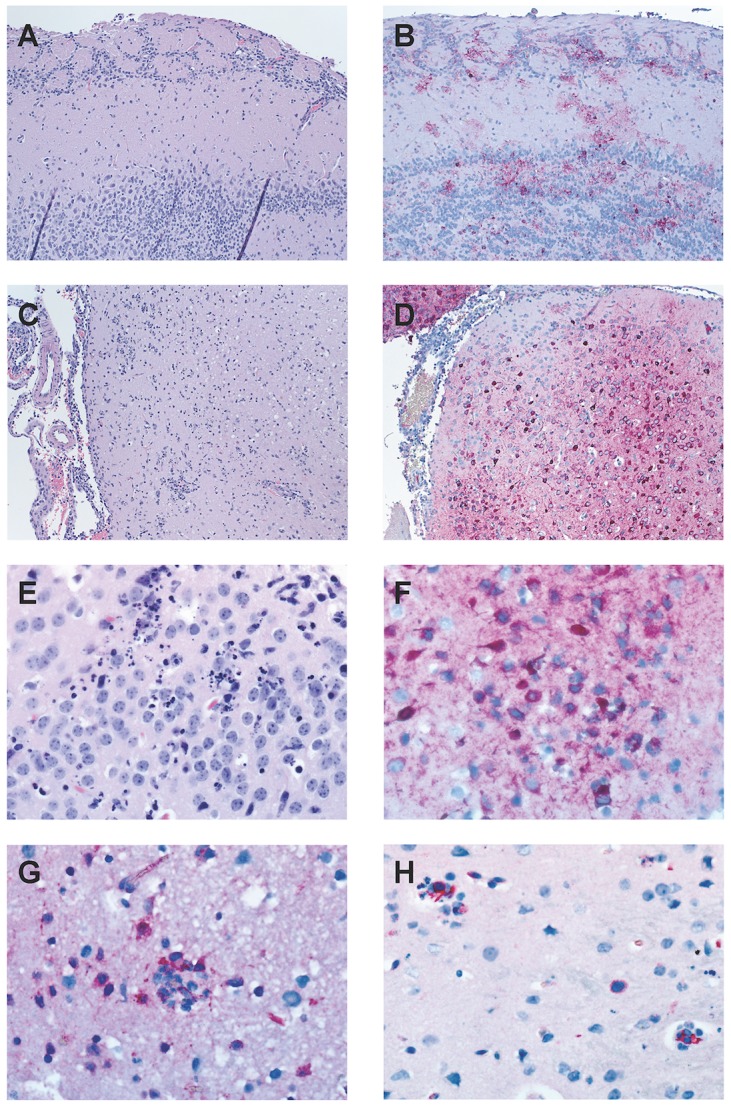
Infection of ΔNSs virus IN causes meningoencephalitis with inflammatory foci characterized by apoptosis and presence of viral antigen, T cells and microglia. Tissues were processed, paraffin-embedded, and sectioned following routine methods, and stained with hematoxylin and eosin (H&E) for histological examination. Immunohistochemistry (IHC) was performed using a monoclonal Rift Valley fever virus antibody to visualize viral antigen and with anti-CD3, anti-CD8, anti-CD20 and anti-CD68 antibodies to identify inflammatory cells. Viral entry appeared to be through the olfactory bulb: (A) H&E and (B) RVFV specific IHC. Meningoencephalitis with multifocal antigen staining is suggestive of subsequent hematogenous spread: (C) subcortical area, H&E and (D) same area, RVFV specific IHC. Inflammatory foci were characterized by (E) morphologic appearance of apoptosis and the presence of (F) RVFV antigen, (G) CD68^+^ microglia, and (H) CD3^+^ cells.

In order to determine the distribution of leukocytes infiltrating the CNS, we evaluated the brains of moribund IN-inoculated mice using histology and IHC. RVFV specific IHC suggested viral entry most likely occurred through olfactory bulbs (Figures 9A and 9B), and multifocal staining throughout the brain was consistent with subsequent vascular and transneural spread of virus. Meningoencephalitis was present and associated with the presence of viral antigen (Figures 9C and 9D). Inflammatory foci were associated with morphologic indications of apoptosis, viral antigen, a CD68^+^ microglial response and the presence of CD3^+^ cells (Figure 9 E-H)

### Depletion of T cells has no effect on mortality following IN infection

**Figure 10:**
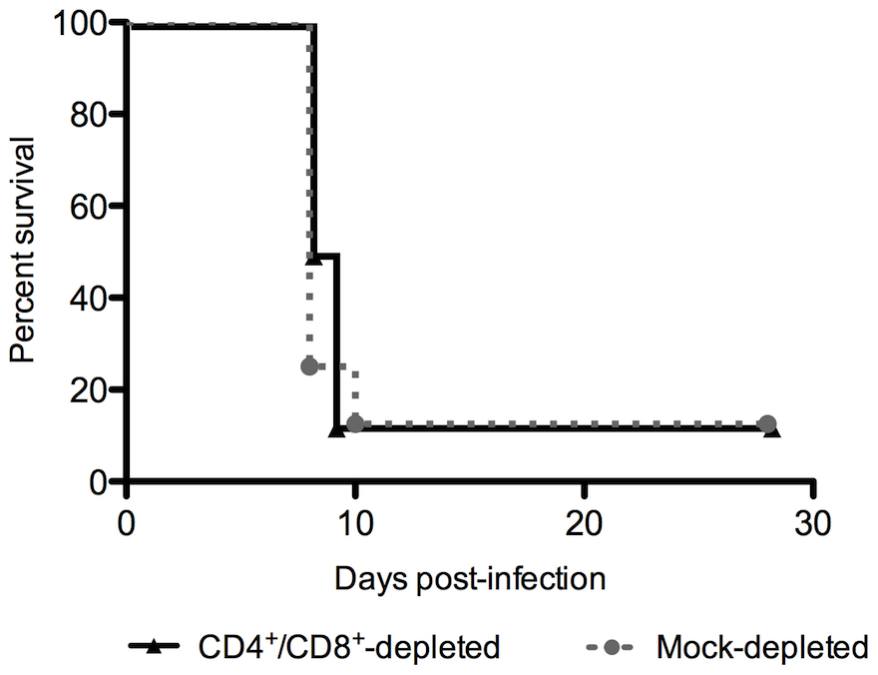
Depletion of T cells did not protect IN mice from death after ΔNSs virus IN infection. Mice were depleted of CD4 and CD8 T cells or mock-depleted on days -3 and -1 prior to infection, and inoculated with 1×10^4^ TCID_50_ ΔNSs virus IN. No significant difference in survival was observed between depleted and mock-depleted mice (n = 10 mice per group).

To explore the possibility that the onset of neurologic disease is dependent on immune-mediated pathology, we depleted mice of CD4^+^ and CD8^+^ T cells prior to ΔNSs IN infection. However, no differences were observed in terms of clinical signs, mortality, or time to death between CD4/CD8-depleted mice and mock-depleted mice (Figure 10).

## Discussion

Encephalitis is a serious and occasionally fatal complication of human RVFV infection, however, little is known about the factors contributing to this disease outcome. There are currently no approved vaccines for human use and no treatment exists beyond supportive care. Development of efficacious preventative and therapeutic measures requires a more detailed understanding of disease pathogenesis, including the kinetics of virus replication and spread, the modes of neuroinvasion, and the protective and potentially pathologic roles of the host immune response. These studies are ideally approached by comparing data from a fatal mouse model of RVFV encephalitis with data from a nonfatal RVFV infection in mice. Here, we demonstrate that ΔNSs virus intranasal inoculation consistently caused fatal neurologic disease 7–9 dpi, a finding that stands in stark contrast to the absence of clinical disease following subcutaneous administration of the virus. Similarly, route-dependent clinical disease was recently described in a mouse model of Hendra virus; mice inoculated parenterally did not develop clinical signs but fatal encephalitis developed following intranasal exposure [22]. Our results also correlate well with those from recent studies that demonstrated enhanced neurovirulence of wild-type RVFV when administered as an aerosol in nonhuman primates [36] and rodents [24], [37].

Comparison of immunocompetent mice inoculated FP or IN permitted characterization of the viral kinetics and immune responses associated with nonfatal and fatal outcomes, respectively. Interestingly, viral RNA loads were similar in peripheral tissues by 2–3 dpi, presumably due to hematogenous spread from the initial sites of virus replication. However, the route of inoculation had a dramatic effect on the quality of the systemic host response; FP-inoculated mice mounted a more rapid and effective immune response than IN-inoculated mice. A robust humoral response, as measured by significantly earlier and higher anti-RVFV IgG and neutralizing antibodies, was associated temporally with early viral clearance from peripheral tissues. Later in infection, FP-inoculated mice initiated a strong T cell response, with significantly higher production of IFNγ, TNFα and IL-2 than IN-inoculated mice. Studies of other encephalitis-associated viruses have shown that IFNγ and TNFα can play a critical role in controlling viral replication in the CNS. For example, in a Japanese encephalitis virus (JEV) model, IFNγ knock-out mice had higher virus RNA titers in brain than wild-type mice, but peripheral tissues have similar viral RNA loads [38], a pattern similar to that seen in our RVFV study, where production of IFNγ was associated with better control of virus. Similarly, following peripheral infection with herpes simplex virus, production of TNF protected mice from development of severe encephalitis [39]. These results suggested that FP inoculation, but not lethal IN inoculation, stimulated a highly effective adaptive immune response that supported viral clearance and prevented progression to clinical disease.

Paradoxically in IN-infected mice, very early in infection there was evidence of enhanced activation of T cells as compared with FP-inoculated mice. These mice produced significant levels of IL-2, a cytokine required for the proliferation and differentiation of T cells (as reviewed in [40]) and enhanced natural killer cell activity [41], that correlated temporally with a marked increase in the proportion of T cells expressing the activation marker CD69. However, in contrast to FP-inoculated mice, the T cells of IN-inoculated mice did not produce IFNγ or TNFα, cytokines associated with the initiation of an appropriate adaptive response and subsequent viral control. The presence of activated T cells that lack effector function has been described previously in models of other neurotropic viruses, including lymphocytic choriomeningitis virus (LCMV) [42] and rabies virus [43]. CNS infection with a highly neurotropic strain of rabies virus resulted in increased expression of CD69 on T cells, but reduced production of inflammatory cytokines IL-2, TNFα and IFNγ. The authors concluded that the apparent T cell ‘unresponsiveness’ resulted from virally-induced immunosuppression specifically targeting the CD4^+^ helper T cell response [43]. The effect of route of infection on CD4^+^ helper T cell function during RVFV infection remains to be evaluated in further detail.

The profound effect of the route of inoculation on host immune response and clinical outcome may be explained, at least in part, by differences in the nature of the immune cells encountered following the initial inoculation. After FP infection, virus immediately encounters resident dendritic (Langerhans) cells and macrophages of the skin. These cells are professional antigen-presenting cells (APCs), and upon recognition of viral antigen, migrate to the draining lymph node to stimulate an early immune response. In contrast, intranasal inoculation resulted in rapid infection in the CNS. There are no detectable dendritic cells in the CNS and microglia are considered poor APCs and limited in the ability to initiate an adaptive response (reviewed in [44]). The delay between initial IN inoculation and systemic infection, coupled with the immunologically privileged nature of the CNS, may help explain the lagging immune response and subsequently enhanced viral replication in IN-inoculated mice.

Viral RNA loads were significantly higher in the brains of IN- relative to FP-inoculated mice, despite similar virus RNA loads in peripheral tissues, suggesting that the timing and mechanism of neuroinvasion were route-dependent. When administered IN, the virus rapidly infected the olfactory bulb and spread caudally to the cerebrum and cerebellum. Multifocal distribution of viral antigen was apparent, suggesting hematogenous and transneural spread from the olfactory bulb. Indeed, active RVFV infection of neuroepithelium and the olfactory bulb have been recently reported [24], strongly supporting neuroinvasion through the olfactory tract in intranasally exposed mice. On the other hand, FP-inoculated mice had virus RNA-positive sciatic nerves 2 dpi, with virus detected several days later in the CNS, and particularly the brainstem, suggesting ΔNSs virus might utilize retrograde transport to access the CNS. Infection of the peripheral nerves of IN-inoculated mice coincided with increasing virus RNA loads in the brainstem, suggesting that axonal virus transport might also occur in the form of anterograde transport.

In light of the apparent hematogenous spread of virus and the presence of cellular infiltrate in the CNS, we evaluated the possibility that virally induced alterations in blood brain barrier permeability led to the onset of clinical disease. In models of JEV [45] and Venezuelan equine encephalitis virus (VEEV) [46] development of viral encephalitis has been linked to inflammatory-mediated alterations in BBB permeability either as a primary mechanism of neuroinvasion or as a mediator of immunopathology. Late stage RVFV has been associated with an increase in proinflammatory cytokines and chemokines [47], Furthermore, in this study, upregulation of IL-6, TNFα, CCL2 and CCL5 gene expression in the brains of IN-inoculated mice was associated with high virus RNA loads and infiltration of large numbers of aberrantly activated CD4^+^ and CD8^+^ T cells. However, to our surprise we found no indication of increased BBB permeability in the mice, even at the height of the CNS inflammatory response, indicating that BBB disruption is not required for RVFV pathogenesis.

Although there were no overt alterations in BBB permeability, it was possible that T cell infiltration and associated upregulation of inflammatory cytokines alone might have enhanced neurovirulence, as seen in other models of viral encephalitis [26], [27], [48]. Induction of these cytokines and chemokines amplify the antiviral response and recruit leukocytes, but if left unchecked, can have destructive effects. In the brains of moribund IN-inoculated mice, a diffuse meningoencephalitis was present and characterized by apoptosis in the presence of viral antigen, T cells and CD68^+^ microglia. To determine if the T cells played a pathologic role in the development of neurologic disease, we depleted mice of both CD4^+^ and CD8^+^ T cells prior to ΔNSs virus intranasal infection. Strikingly, we observed no improvement in survival of T cell-depleted mice relative to mock-depleted mice. These results indicate that the development of RVFV encephalitic disease is not T cell-mediated, and instead suggests that direct viral damage is responsible for the onset of severe disease.

In summary, we developed a mouse model of fatal RVFV encephalitic disease utilizing intranasal inoculation with an attenuated RVFV. Fatal human cases of RVFV encephalitis are relatively uncommon, and there are few descriptions of the CNS pathology. However, the available case reports from South Africa and Egypt describe focal areas of necrosis with perivascular cuffing [49] and degeneration of the cerebral neurons with infiltration of microglial cells [50]. Although these findings are fairly nonspecific, they are similar to the lesions seen in the intranasally infected mice, suggesting this is a reasonable model of RVFV encephalitis. Comparison of the course of disease following IN or FP inoculation of ΔNSs virus highlighted the potential roles of the host immune response in determining clinical outcome. IN inoculation resulted in rapid and fulminant virus replication in the CNS via infection of the olfactory bulb, whereas FP-inoculated mice had low virus RNA loads in the CNS and survived without developing disease. Despite different outcomes, peripheral virus kinetics were the same following both routes of inoculation, until viral clearance, associated with a robust antibody response, occurred exclusively in FP-inoculated mice. IN-inoculated mice displayed an inappropriate T cell response; although apparently activated, T cells failed to release critical cytokines for control of virus replication. The ineffective adaptive immune responses of IN-inoculated mice were associated with the development of peak virus RNA loads, strong proinflammatory responses and infiltration of activated T cells into the CNS, followed by severe clinical disease and death. Interestingly, in contrast to other encephalitic viruses, T cells were not required for development of neurologic disease, suggesting that RVFV encephalitis is not immune-mediated. Although we cannot exclude a role for inflammatory cytokines and other immune cells in the development of clinical disease, our studies strongly point to direct viral destruction of the brain as the major cause. These findings have implications for the development of therapeutics targeting RVFV neurologic disease, which may be particularly useful given the relatively delayed onset of RVFV encephalitis in infected humans.
